# Dual trigger of triptorelin and HCG optimizes clinical outcome for high ovarian responder in GnRH-antagonist protocols

**DOI:** 10.18632/oncotarget.23916

**Published:** 2018-01-04

**Authors:** Saijiao Li, Danni Zhou, Tailang Yin, Wangming Xu, Qingzhen Xie, Dan Cheng, Jing Yang

**Affiliations:** ^1^ Reproductive Medicine Center, Renmin Hospital of Wuhan University, Hubei Clinic Research Center for Assisted Reproductive Technology and Embryonic Development, Wuhan, Hubei 430060, P.R. China

**Keywords:** dual trigger, GnRH agonist, GnRH antagonist protocols, OHSS, high ovarian response

## Abstract

In this paper, a retrospective cohort study was conducted to the high ovarian responders in GnRH-antagonist protocols of IVF/ICSI cycles. The purpose of the study is to investigate whether dual triggering of final oocyte maturation with a combination of gonadotropin-releasing hormone (GnRH) agonist and human chorionic gonadotropin (HCG) can improve the clinical outcome compared with traditional dose (10000IU) HCG trigger and low-dose (8000IU) HCG trigger for high ovarian responders in GnRH-antagonist *in vitro* fertilization/intracytoplasmic sperm injection (IVF-ICSI) cycles. Our study included 226 couples with high ovarian responders in GnRH-antagonist protocols of IVF/ICSI cycles. Standard dosage of HCG trigger (10000 IU of recombinant HCG) versus dual trigger (0.2 mg of triptorelin and 2000 IU of recombinant HCG) and low-dose HCG trigger (8000IU of recombinant HCG) were used for final oocyte maturation. Our main outcome measures were high quality embryo rate, the number of usable embryos, the risk of OHSS, duration of hospitalization and incidence rate of complications. Our evidence demonstrated that dual trigger is capable of preventing severe OHSS while still maintaining excellent high quality embryo rate in in high ovarian responders of GnRH-antagonist protocols.

## INTRODUCTION

Over the past 2 decades, gonadotrophin-releasing hormone (GnRH) antagonist protocols has been proposed as a safer and efficacious way for ovarian stimulation [1]. GnRH antagonists protocols have several advantages over the long agonist, including the rapid decrease luteinizing hormone (LH) and follicle-stimulating hormone (FSH) levels, without flare-up effect, a lower consumption of used gonadotropins, shorter overall treatment duration [2], especially for reducing the incidence of ovarian hyper-stimulation syndrome (OHSS) [3, 4].

Recently, GnRH agonist (GnRH-a) have been used to trigger final oocyte maturation after ovarian stimulation. Earlier studies have concluded that the use of a GnRH-a trigger has equal oocyte yield, maturity, fertilization, implantation, pregnancy compared to human chorionic gonadotropin (HCG) trigger in GnRH antagonist protocols [5]. An important advantage of using a GnRH-a to trigger oocyte maturation is the induction of a more physiologic release of LH and FSH, which is similar to the natural menstrual cycle [6, 7], thus provides an alternative to the administration of HCG in protocols. In addition, some studies have reported retrieval of more mature oocytes, optimize the pregnancy outcomes, and may reduce the incidence of immature oocyte syndrome for a sub-group of women, when triggered with GnRH-a compared with HCG [8–10].

“Dual trigger” was first defined as the concept of a combination of GnRH-a and a low-dose HCG in triggering final oocyte maturation [11], primarily for the prevention of OHSS in high ovarian responders. As well as the endogenous LH and FSH released by the GnRH-a and low-dose of HCG, would result in more mature oocytes, optimization of live birth rates in high ovarian responders compared with the traditional dose (10000IU) HCG trigger alone [9, 12–13]. As an innovation in our study, we divided the HCG trigger group into two subgroups, according to the dosage of HCG, the low-dose (8000IU) HCG trigger and the traditional dose (10000IU) HCG trigger. The low-dose HCG trigger has a better effect in preventing the high risk of OHSS than the traditional dose HCG trigger in the high ovarian responders. The data analysis among the three groups about clinical outcomes is more convictive.

We therefore explored the use of dual trigger of oocyte maturation with GnRH-a and low-dose HCG (2,000 IU) in high ovarian responders of GnRH-antagonist protocols. The aim of the study was to compare the number of retrieved oocytes, fertilization rate, high qualified embryo rate and valuate the OHSS risk after dual GnRH-a and low-dose HCG trigger versus HCG trigger (10000IU or 8000IU) alone.

## RESULTS

### General information

In this patient cohort, the three groups were compared for age, BMI, infertility causes, duration of infertility, baseline FSH and E_2_, duration of gonadotropin (Gn) stimulation, and Gn dose received by the patients. No significant differences were observed for any of the parameters (P > 0.05) (Table 1).

**Table 1:** General information of three groups

	Control	Low-dose HCG	Dual trigger	P value
n.	98	85	43	
Age of women (y)	29.21±4.35	28.66±3.31	28.77±4.01	ns.
BMI of women (kg/m^2^)	22.29±2.62	22.09±3.55	22.43±3.05	ns.
Infertility causes, n (%)				
Tubal factor, n (%)	38(38.78)	35(41.18)	24(55.81)	ns.
Male factor, n (%)	11(11.22)	9(10.59)	1(2.33)	
PCOS, n (%)	36(36.73)	32(37.65)	16(37.21)	
Combination of factors	13(13.27)	9(10.59)	2(4.65)	
Duration of infertility (y)	3.40±3.06	3.12±2.67	2.65±1.33	ns.
Baseline FSH (IU/L)	6.20±1.58	6.22±1.36	6.18±1.45	ns.
Baseline E_2_ (pg/ml)	45.32±17.80	46.81±14.53	49.42±16.51	ns.
Baseline LH (mIU/mL)	5.83±3.79	5.84±3.27	6.18±3.58	ns.
Duration of Gn (days)	10.11±1.74	9.93±1.74	9.91±1.53	ns.
Starting Gn dose (IU)	164.28±40.81	165.60±44.60	166.02±50.37	ns.
Total Gn ampoules (75 IU)	23.53±7.61	23.94±7.95	24.50±7.60	ns.

All continuous variables are expressed as ‘Mean±SD’.

### Embryological data

The mean E_2_ concentration on the day of HCG administration, number of oocytes retrieved, number of oocytes retrieved and quality embryo rate were also comparable among the three groups. Due to the high risk of OHSS, patients with higher E_2_ concentration on the day of HCG trends to use lower dose of HCG to trigger in high ovarian responders. The mean E_2_ concentration on the day of HCG administration, numbers of oocytes retrieved were significantly increased in control group. High quality embryo rate and the number of usable embryos was significant decreased in control group (P < 0.05) (Table 2). No significant differences were observed in fertilization rate, cleavage rate of patients (P > 0.05). The parameters of low-dose HCG group and dual trigger group are similar, and there is no statistically significant difference between them.

**Table 2:** Cycle parameters and embryological data of the three groups

	Control	Low-dose HCG	Dual trigger	P^a^	P^b^
n.	98	85	43		
E_2_ on the day of HCG (pg/ml)	5115.14±942.68	6932.53±2082.35	6824.70±2718.04	.000	.000
No. oocytes retrieved	18.54±4.38	20.27±5.42	21.63±7.43	.002	.034
Fertilization rate (%)	68.82±19.49	70.81±17.10	64.86±18.67	ns.	ns.
Cleavage rate (%)	96.69±6.62	97.00±.5.14	97.33±4.75	ns.	ns.
Quality embryo rate (%)	52.83±23.71	59.60±19.37	64.21±22.34	.005	.038
No. usable embryos (n)	10.19±4.61	12.25±5.28	13.58±7.21	.001	.011

^a^ Difference between Low-dose HCG and Control group. P value <0.05 was considered statistically significant.

^b^ Difference between Dual trigger and Control group. P value <0.05 was considered statistically significant.

All continuous variables are expressed as means ‘Mean±SD’.

### Clinical outcomes and OHSS risk

All 226 patients were followed up until their next menses. The clinical outcomes of patients in three groups are shown in Table 3. Only 5 patients had quite severe OHSS which requiring abdominal paracentesis, and all of them are from the low-dose HCG group. The duration of hospitalization was 5.55±1.82 days in the control group, 5.98±2.99 days in the low-dose HCG group, 5.02±1.51 days in the dual trigger group. The dual trigger group has a lower duration of hospitalization than any others (P<0.05). The proportion of patients at high risk for OHSS who developed moderate early OHSS was 32.65 %, 41.18% and 39.53% in the control group, low-dose HCG group and dual trigger group, respectively. The incidence of severe early OHSS in the patients was 2.04% (2/98), 9.41% (8/85) and 2.34% (1/43) in the control group, low-dose HCG group and dual trigger group, respectively. In our studies, the main complications of OHSS are liver damage or have a fever, the incidence rate of complication is the most highest in the low-dose HCG group. In Table 3, it demonstrated that the risk of OHSS, duration of hospitalization and incidence rate of complications were lower in dual trigger group than the other two groups (P < 0.05).

**Table 3:** OHSS outcomes of high-risk treatment and control groups

	Control group	Low-dose HCG	Dual trigger	X^2^	P
n.	98	85	43		
Paracentesis, n (%)	0	5(5.88)	0	8.627	.013
Length of hospital stay (days)	5.55±1.82	5.98±2.99	5.02±1.51^c^		.029
Severity of OHSS					
Mild, n (%)	56(57.14)	37(43.53)	17(39.53)	15.279	.018
Moderate, n (%)	32(32.65)	35(41.18)	17(39.53)		
Severe, n (%)	2(2.04)	8(9.41)	1(2.34)		
Complications (n)					
Liver damage, n (%)	2(2.04)	14(16.47)	3(6.98)	13.337	.010
Fever, n (%)	2(2.04)	1(1.18)	0		

^c^ Difference between Low-dose HCG and Dual trigger group, and P <0.05.

P <0.05 was considered statistically significant.

The continuous variable is expressed as ‘Mean±SD’.

## DISCUSSION

GnRH antagonist protocol with the use of GnRH-agonist for final oocyte maturation had emerged as the most effective method of preventing early onset OHSS without cycle cancellation during the past decades [15]. The GnRH-a trigger was applied to GnRH-antagonist protocol, that induces oocyte maturation and prevent the high risk of OHSS [16]. Due to that GnRH-a trigger can cause an abnormal endogenous LH surge, which lasted only 24–36 hours after the GnRH-a trigger [17], leads to the abnormal corpora lutea function [18]. GnRH-a trigger alone is sufficient to induce oocyte maturation, but not to produce the prolonged stimulation of the corpus luteum, compared with the use of traditional dose of HCG trigger [19]. So dual trigger had been recognized, which can maintain the optimal lutealphase function and prevent the high risk of OHSS [11]. The previous study demonstrated that GnRH antagonist protocol with the dual trigger for final oocyte maturation had to prevent hospitalization for OHSS in patients with an exaggerated response to gonadotrophin stimulation, while not compromising pregnancy outcomes [20].

The results from our study investigated that dual-triggered oocyte maturation can be an effective strategy to optimize high quality embryos rate and improve the clinical outcomes of high risk OHSS in high ovarian responders of GnRH-antagonist cycles. Our study results emphasize that the dual trigger statistically significantly improved high quality embryo rate and the clinical outcomes of high risk OHSS compared with others. In spite of several studies in past have been conducted to compare the efficacy of dual trigger with GnRH-a trigger in stimulating ovulation for fresh cycles with prevention of OHSS in high responders [9, 21], our study compared dual trigger with HCG trigger and low-dose HCG trigger in high ovarian responders is likely to be innovated.

In our study, the dual trigger strategy significantly increased the high quality embryo rate, which can be predictive for the assisted reproductive technology outcome. Data from sibling human oocytes suggest that embryo quality improves indicating an improvement of cytoplasmatic maturation [22], so dual trigger for oocyte maturation may optimize the cytoplasmatic maturation in high ovarian responders of GnRH-antagonist protocols.

OHSS is the most dangerous and life-threatening complication associated with ovarian stimulation during assisted reproductive technology [23]. Several strategies have been proposed to prevent or minimize the intensity of OHSS without compromising pregnancy outcome, expert for cycle cancellation, GnRH antagonist protocol is effective in preventing OHSS in high ovarian responders [24]. In the present study, the risk of OHSS, duration of hospitalization and incidence rate of complications were lower in dual trigger group than low dosage of HCG or standard dosage of HCG for high ovarian responder in GnRH-antagonist protocols. According to our research, the dual trigger in GnRH-antagonist protocols can effectively prevent and relive the symptoms of OHSS in high ovarian responders.

Nevertheless, our study demonstrates that dual trigger can effectively prevent the severe OHSS and optimize high quality embryo production in high ovarian responders of GnRH-antagonist protocols. It must also be acknowledged that our data require future investigators conducting large prospective trials on influence of clinical pregnancy outcome. Therefore, larger prospective randomized controlled studies are needed to evaluate the optimal timing and dosage of HCG to be given with GnRH-a trigger to maintain assisted reproductive technology outcome without increasing the risk of clinically significant OHSS.

## MATERIALS AND METHODS

### Study design

The patients from January 1, 2015, through July 31, 2016 were recruited in this retrospective cohort study for all IVF/ICSI cycles with a GnRH-antagonist protocol at the Reproductive Medicine Center in Renmin Hospital of Wuhan University in Wuhan, China. The couples were given counseling regarding the high risks and symptoms of OHSS and all agreed to cancel fresh embryo transfer. Each patient was allowed to participate in the study only once.

### Study participants

A total of 226 high ovarian responders (estradiol level greater than 4,000 pg/mL on the day of triggering or as the number of retrieved oocytes ≥20) completed IVF/ICSI cycles were included for final analysis. The exclusion criteria were advanced reproductive age (≥40 years), severe underweight or overweight status (body mass index, BMI≤18 or ≥25 kg/m^2^), occult ovarian failure (day-3 FSH concentration of ≥8 IU/L), presence of endocrine disorders (diabetes mellitus, hyperprolactinemia, thyroid dysfunction, congenital adrenal hyperplasia, Cushing syndrome), or uterine anomaly confirmed by either hysterosalpingography or hysteroscopy.

The diagnostic criteria for OHSS were according to Golan's classification [14]. Patients with mild OHSS presented with symptoms of mild abdominal distension and discomfort, possibly accompanied by nausea, vomiting and diarrhea, and an ovarian diameter of ≤5 cm. Moderate OHSS was defined as an aggravation of the aforementioned symptoms, associated with a weight gain of ≥4.5 kg, ascites identified by ultrasound examination and an ovarian diameter of 5–10 cm. Severe OHSS was defined as marked ascites and/or hydrothorax, hematocrit≥45%, white blood cell count (WBC) ≥15,000/mm^3^, dyspnea, oliguria or abnormal liver function tests, and large ovaries (≥10 cm maximum diameter). No other intervention for the prevention of OHSS was undertaken, except for the ‘freeze all embryos’ in the event of moderate-to-severe early OHSS.

The choice of either HCG trigger or dual trigger was based on physician preference. GnRH-a (0.2mg) and low-dose HCG (2,000 IU) cycles served as dual trigger group (n =43), compared with the low-dose HCG trigger (8000IU) cycles (low-dose HCG group, n =85) and the traditional HCG trigger cycles (control group, n =98).

### *In vitro* fertilization/intracytoplasmic sperm injection (IVF/ICSI) procedure

The patients underwent ovarian stimulation accomplished with an individualized GnRH antagonist protocol. All patients had a baseline transvaginal ultrasound on menstrual cycle day 2 and started recombinant FSH (Gonal-F, Merck Serono), ranging from 150 to 225 IU was based on patient age, body mass index, day 2 FSH, antral follicle count, and previous response to Controlled Ovarian Hyperstimulation (COH) on the secend day of the menstrual cycle for 3 consecutive days.

Patient response was monitored during the IVF cycle with serial transvaginal ultrasounds for follicular measurements and serum estradiol (E_2_) levels. The dose of gonadotropins was adjusted according to a patient's response. GnRH antagonist (Ganirelix; Organon USA) was started at 0.25 mg subcutaneously daily once a follicle reached ≥14 mm in diameter or serum E_2_ reached ≥200 pg/mL until the day of oocyte maturation trigger. Patients were triggered once three follicles reached ≥17 mm in diameter with either 0.2mg triptorelin acetate (Decapeptyl; Ferring Gmbh, Genmany) subcutaneously and 2,000 IU HCG (Livzon Group, China) intramuscularly or 8000IU-10000IU HCG intramuscularly. Transvaginal ultrasound–guided oocyte retrieval was performed 36 hours after trigger injection. Serum LH, E_2_, progesterone (P), and HCG levels were assessed the day after trigger to ensure adequate LH surge response and HCG absorption. All embryo transfers were performed 72 hours after oocyte retrieval. The remaining viable embryos were cultured to the blastocyst stage and were cryopreserved by vitrification.

### Monitoring of patients

Monitoring of patients consisted of general information, symptoms, complications during the hospitalization (ovarian torsion, thromboembolic events), embryonic condition, BMI, abdomen circumference, ascites, and pleural effusion, urine output, days of luteal phase, whether paracentesis had taken place, and the amount of albumin (Alb) transfused were monitored and recorded. Biochemical values such as hematocrit, white blood cell count, Alb levels, blood urea nitrogen, creatinine, liver enzymes, prothrombin time, and partial thrombin time were measured when necessary.

### Statistical analysis

Statistical analysis was performed using SPSS 13.0 statistical software (Chicago, IL, U.S.) according to the intention to treat principle. Chi-square or Fisher exact tests were used for categorical l variables, and independent-sample t test was used for continuous variables where appropriate. Data are presented as Mean±SD. All analyses of significance were two-sided and tested at the 5 % level. P < 0.05 was considered statistically significant. Continuous variables were tested if they presented normal distribution using the F-test. The results of the multiple groups were compared using the ANOVA and the comparison among groups was performed with an LSD test. Qualitative variables were assessed with the chi-squared method and Yate's correction.

### Declarations sections

#### Ethical approval and consent to participate

We strictly obeyed the Declaration of Helsinki for Medical Research involving human subjects during the project and written consent was obtained from all subjects. The study protocol was approved by the medical ethics committee and institutional review board of Renmin Hospital of Wuhan University.

#### Consent for publication

Written informed consents for the publication of related Tables and figures had been obtained from the individuals.

#### Availability of supporting data

All the data is contained in the manuscript.

## CONCLUSION

In conclusion, our study suggests that dual trigger of GnRH-a and low-dose HCG (2,000 IU) could improve the reproductive outcome (higher quality embryo rate) and prevent severe OHSS among the high ovarian responders in GnRH-antagonist protocols, compared to GnRHa trigger or HCG trigger alone. Prior to the dual trigger's routine implementation, further large prospective studies are needed to examine the role of dual triggers in the high ovarian responders in GnRH-antagonist protocols. Dual trigger should be advocated as a safe approach to optimizing clinical outcome without cycle cancelled in high ovarian responders of GnRH-antagonist protocols.

## Abbreviations

Alb: amount of albumin
BMI: body mass index
E_2_: estradiol
FSH: follicle-stimulating hormone
Gn: gonadotropin
GnRH: gonadotropin-releasing hormone
GnRH-a: gonadotropin-releasing hormone agonist
HCG: human chorionic gonadotropin
IVF-ICSI: *in vitro* fertilization/intracytoplasmic sperm injection
LH: luteinizing hormone
OHSS: ovarian hyper-stimulation syndrome
P: progesterone
WBC: white blood cell count.

## References

[1] Garcia-Velasco JA, Fatemi HM (2015). To pill or not to pill in GnRH antagonist cycles: that is the question!. Reprod Biomed Online.

[2] Devroey P, Aboulghar M, Garcia-Velasco J, Griesinger G, Humaidan P, Kolibianakis E, Ledger W, Tomás C, Fauser BC (2009). Improving the patient's experience of IVF/ICSI: a proposal for an ovarian stimulation protocol with GnRH antagonist co-treatment. Hum Reprod.

[3] Al-Inany HG, Abou-Setta AM, Aboulghar M (2007). Gonadotrophin-releasing hormone antagonists for assisted conception: a Cochrane review. Reprod Biomed Online.

[4] Mokhtar S, Sadeghi MR, Akhondi MM, Zafardoust S, Badenush B, Fatemi F, Nazari F, Kamali K, Mohammadzade A (2015). ART outcomes in GnRH antagonist protocol (flexible) and long GnRH agonist protocol during early follicular phase in patients with polycystic ovary syndrome: a randomized clinical trial. J Reprod Infertil.

[5] Gustofson RL, Surrey ES, Minjarez DA, Schoolcraft W (2011). GnRH-agonist (GnRH-A) trigger in GnRH-antagonist cycles compared to human chorionic gonadotropin (hCG) trigger in long luteal leuprolide downregulation (LL) cycles for hyperresponding oocyte donors. Fertil Steril.

[6] Humaidan P, Kol S, Papanikolaou EG (2011). Copenhagen GnRH agonist triggering workshop group. GnRH agonist for triggering of final oocyte maturation: time for a change of practice?. Hum Reprod Update.

[7] Griesinger G, Diedrich K, Devroey P, Kolibianakis EM (2005). GnRH agonist for triggering final oocyte maturation in the GnRH antagonist ovarian hyperstimulation protocol: a systematic review and meta-analysis. Hum Reprod Update.

[8] Humaidan P, Ejdrup Bredkjær H, Bungum L, Bungum M, Grøndahl ML, Westergaard L, Yding Andersen C (2005). GnRH agonist (buserelin) or hCG for ovulation induction in GnRH antagonist IVF/ICSI cycles: a prospective randomized study. Hum Reprod.

[9] Griffin D, Benadiva C, Kummer N, Budinetz T, Nulsen J, Engmann L (2012). Dual trigger of oocyte maturation with gonadotropin-releasing hormone agonist and low-dose human chorionic gonadotropin to optimize live birth rates in high responders. Fertil Steril.

[10] Griffin D, Engmann L, Budinetz T, Kummer N, Nulsen J, Benadiva C (2012). Dual trigger with gonadotropin releasing hormone agonist (GnRHa) and human chorionic gonadotropin (hCG) for the treatment of “immature oocyte syndrome” (IOS). Fertil Steril.

[11] Shapiro BS, Daneshmand ST, Garner FC, Aguirre M, Thomas S (2008). Gonadotropin-releasing hormone agonist combined with a reduced dose of human chorionic gonadotropin for final oocyte maturation in fresh autologous cycles of in vitro fertilization. Fertil Steril.

[12] Griffin D, Feinn R, Engmann L, Nulsen J, Budinetz T, Benadiva C (2014). Dual trigger with gonadotropin-releasing hormone agonist and standard dose human chorionic gonadotropin to improve oocyte maturity rates. Fertil Steril.

[13] Castillo JC, Morenoa J, Dolza M, Bonilla-Musoles F (2013). Successful pregnancy following dual triggering concept (rhCG + GnRH agonist) in a patient showing repetitive inmature oocytes and empty follicle syndrome: case report. J Med Cases.

[14] Golan A, Ron-el R, Herman A, Soffer Y, Weinraub ZC (1989). Ovarian hyperstimulation syndrome: an update review. Obstet Gynecol Surv.

[15] Datta AK, Eapen A, Birch H, Kurinchi-Selvan A, Lockwood G (2014). Retrospective comparison of GnRH agonist trigger with HCG trigger in GnRH antagonist cycles in anticipated high-responders. Reprod Biomed Online.

[16] Itskovitz-Eldor J, Kol S, Mannaerts B (2000). Use of a single bolus of GnRH agonist triptorelin to trigger ovulation after GnRH antagonist ganirelix treatment in women undergoing ovarian stimulation for assisted reproduction, with special reference to the prevention of ovarian hyperstimulation syndrome: preliminary report: short communication. Hum Reprod.

[17] Damewood MD, Shen W, Zacur HA, Schlaff WD, Rock JA, Wallach EE (1989). Disappearance of exogenously administered human chorionic gonadotropin. Fertil Steril.

[18] Ashraf A, Shayesteh M, Marzieh G (2016). GnRH agonist trigger versus hCG trigger in GnRH antagonist in IVF/ICSI cycles: a review article. Int J Reprod Biomed.

[19] Itskovitz J, Boldes R, Levron J, Erlik Y, Kahana L, Brandes JM (1991). Induction of preovulatory luteinizing hormone. Fertil Steril.

[20] Radesic B, Tremellen K (2011). Oocyte maturation employing a GnRH agonist in combination with low-dose hCG luteal rescue minimizes the severity of ovarian hyperstimulation syndrome while maintaining excellent pregnancy rates. Hum Reprod.

[21] de Oliveira SA, Calsavara VF, Cortés GC (2016). Final oocyte maturation in assisted reproduction with human chorionic gonadotropin and gonadotropin-releasing hormone agonist (dual trigger). JBRA Assist Reprod.

[22] Hassan HA (2001). Cumulus cell contribution to cytoplasmic maturation and oocyte developmental competence in vitro. J Assist Reprod Genet.

[23] Pfeifer S, Butts S, Dumesic D, Fossum G, Gracia C, La Barbera A, Mersereau J, Odem R, Paulson R, Penzias A, Pisarska M, Rebar R, Reindollar R (2016). Prevention and treatment of moderate and severe ovarian hyperstimulation syndrome: a guideline. Fertil Steril.

[24] Fouda UM, Sayed AM, Elshaer HS, Hammad BE, Shaban MM, Elsetohy KA, Youssef MA (2016). GnRH antagonist rescue protocol combined with cabergoline versus cabergoline alone in the prevention of ovarian hyperstimulation syndrome: a randomized controlled trial. J Ovarian Res.

